# Autophagy in Virus Infection: A Race between Host Immune Response and Viral Antagonism

**DOI:** 10.3390/immuno2010012

**Published:** 2022-01-30

**Authors:** Karan Chawla, Gayatri Subramanian, Tia Rahman, Shumin Fan, Sukanya Chakravarty, Shreyas Gujja, Hayley Demchak, Ritu Chakravarti, Saurabh Chattopadhyay

**Affiliations:** 1 Department of Medical Microbiology and Immunology, University of Toledo College of Medicine and Life Sciences, Toledo, OH 43614, USA; 2 Department of Physiology and Pharmacology, University of Toledo College of Medicine and Life Sciences, Toledo, OH 43614, USA

**Keywords:** autophagy, innate immunity, anti-viral response, pro-viral response, viral antagonism

## Abstract

Virus-infected cells trigger a robust innate immune response and facilitate virus replication. Here, we review the role of autophagy in virus infection, focusing on both pro-viral and anti-viral host responses using a select group of viruses. Autophagy is a cellular degradation pathway operated at the basal level to maintain homeostasis and is induced by external stimuli for specific functions. The degradative function of autophagy is considered a cellular anti-viral immune response. However, autophagy is a double-edged sword in viral infection; viruses often benefit from it, and the infected cells can also use it to inhibit viral replication. In addition to viral regulation, autophagy pathway proteins also function in autophagy-independent manners to regulate immune responses. Since viruses have co-evolved with hosts, they have developed ways to evade the anti-viral autophagic responses of the cells. Some of these mechanisms are also covered in our review. Lastly, we conclude with the thought that autophagy can be targeted for therapeutic interventions against viral diseases.

## 1. Introduction

Virus infection triggers a robust cellular immune response via activating the pattern recognition receptors [1-3]. The innate immune response is critical to ensure an immediate anti-viral response and also to help shape the adaptive immune responses [4,5]. In addition to triggering the anti-viral signaling pathways, the virus-infected cells can induce autophagy in the infected cells [6,7]. Autophagy, which can be translated literally as “self-eating”, is a cellular catabolic process required to regulate many cellular functions [8]. Basal level autophagy is required for maintaining cellular homeostasis, and autophagy is often induced in various disease conditions and infections [9]. The catabolic property of autophagy is primarily utilized as a cellular immune response to inhibit viral replication by degrading viral proteins. However, many viruses often exploit autophagy to replicate effectively within infected cells [10]. Therefore, a careful balance between the pro-viral and anti-viral functions of autophagy is important. It is also evident that viruses that do not rely on the machinery of autophagy for replication can still benefit from the components of the pathway. Moreover, autophagy pathway components can participate in autophagy-independent cellular functions. The therapeutic targeting of autophagy pathway components may provide clinical options for the treatment of viral and non-viral diseases [11,12].

## 2. Autophagy: Activation and Various Forms

### 2.1. Cellular Autophagy Pathway

**Figure 1.**
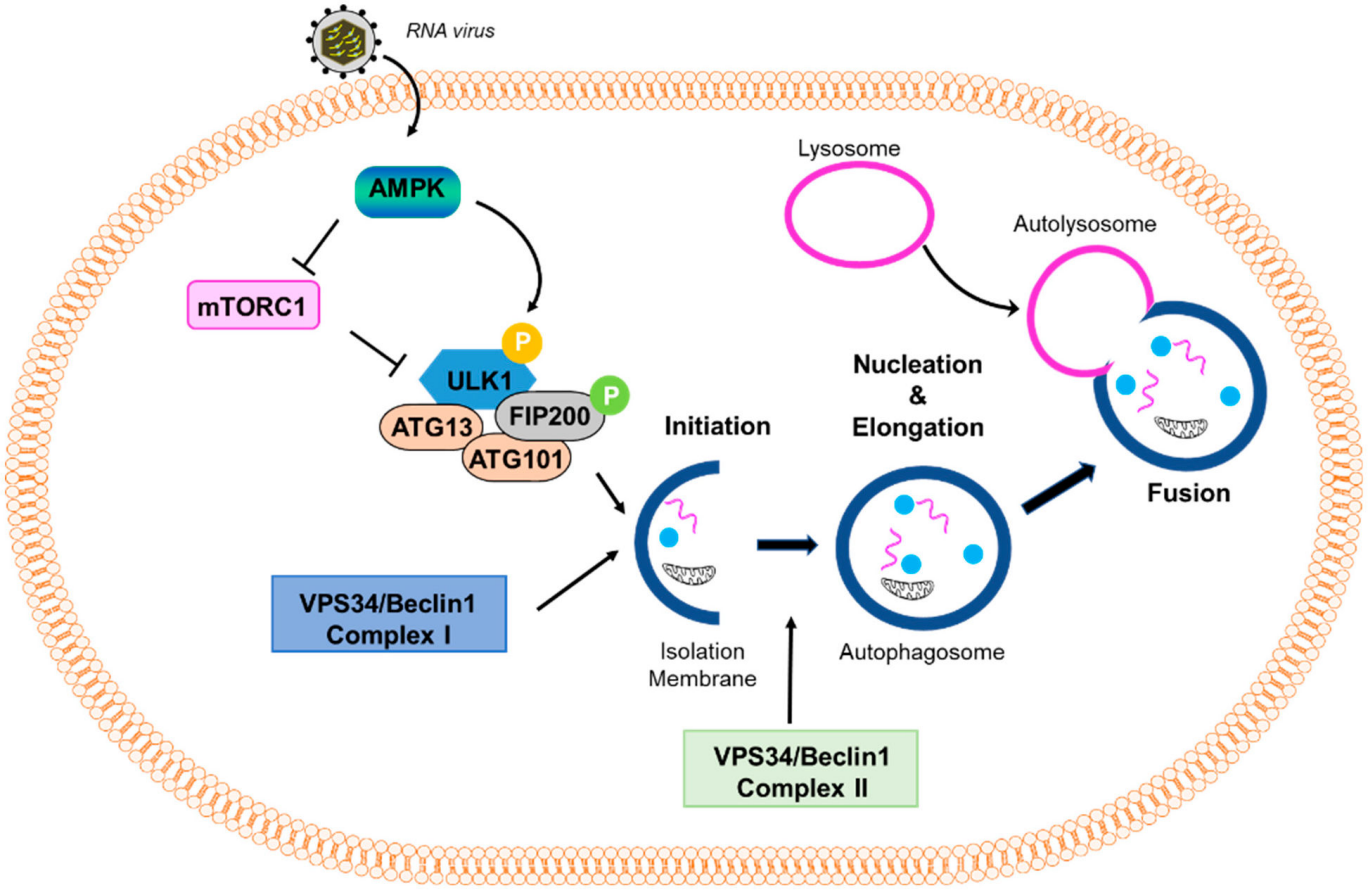
Virus-induced autophagy pathway. A scheme of the autophagy pathway: mTOR inhibits the autophagy pathway by negatively regulating the downstream effector protein ULK1 through phosphorylation. Stressful conditions, such as virus infection, activate AMPK, which inhibits mTOR and promotes the activation of ULK1. Activation of downstream effectors, such as ULK1 and VPS34/Beclin1 Complex I, induce the formation of an isolation membrane and initiate the formation and maturation of the autophagosome. VPS34/Beclin1 Complex II is important in the formation of the autophagosome. The autophagosome then fuses with the lysosome to form the autolysosome.

**Figure 2.**
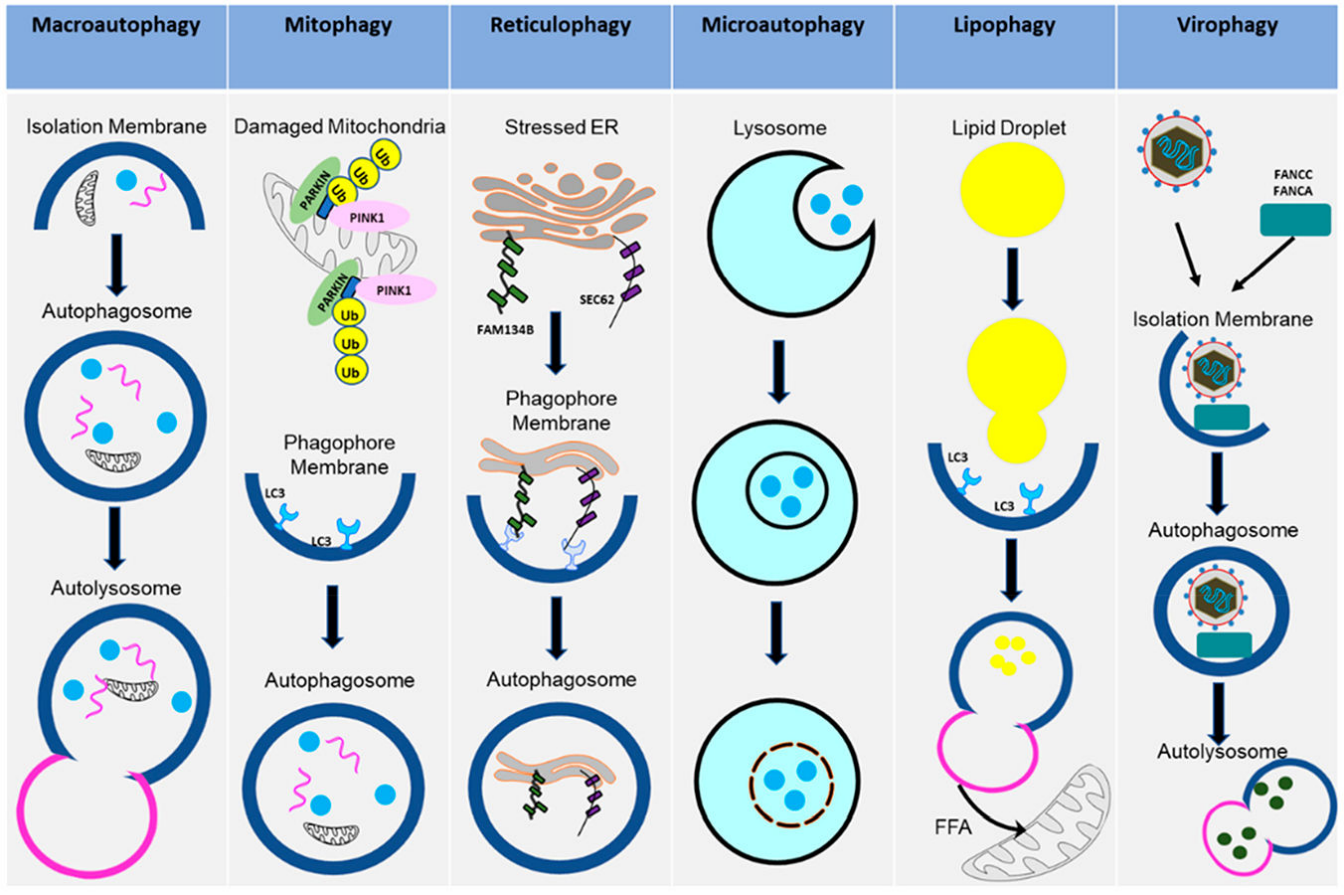
Various types of autophagy pathways. The diagram shows how the cells activate different types of autophagy pathways in the uninfected or the virus-infected cells. These pathways contribute to cellular homeostasis or disease states.

Macroautophagy (referred to in this paper as autophagy from this point on), as the name suggests, signifies self- (*auto*) eating (*phago*), and serves as a cellular degradative process. Autophagy occurs at a basal level in healthy cells but is also activated in response to stressors, such as nutrient deprivation, organelle damage, oxidative burst, and viral infection. Autophagy is a cellular pathway that involves four major steps: initiation, nucleation, elongation, and fusion, as depicted in Figure 1. The various forms of autophagy, as detailed below, are shown in Figure 2.

Autophagy is induced when AMP-dependent kinase (AMPK) is activated in response to stressors, leading to the downstream phosphorylation and activation of ULK1 as well as the inhibition of mTOR [13]. ULK1 functions in a complex with the additional proteins Fip200, Atg101, and Atg13. This complex leads to the activation of the PI3K complex, which comprises Beclin-1, Vps34, and Vps15. The activated PI3K complex converts PIP2 to PIP3, which results in the recruitment of human WD-repeat protein interacting with the phosphoinositide (WIPI) proteins, WIPI1 and WIPI2. The WIPI proteins play an essential role in recognizing and reading the PtdIns3P signal at the nascent autophagosome. Thus, the nucleation stage of autophagy begins, during which the phagophore starts to form. The phagophore is a double-membraned vacuole that encapsulates the proteins to be recycled or degraded, along with other cytoplasm-derived proteins. Next, the elongation process is an Atg5-Atg12-Atg16-dependent step that results in the formation of an autophagosome, which is marked by the lipidation of LC3-I to LC3-II and involves Atg7 and Atg3. After elongation of LC3, the autophagosome fuses with a lysosome using the additional proteins, SNARE and Rab7, which form a structure called the autophagolysosome. Lastly, acidification of the contents within the autophagolysosome occurs, resulting in the degradation of the protein content. This step is marked by the degradation of p62, also known as sequestome-like receptor (SLR), the autophagic adaptor, which binds and packages the cargo into the autophagolysosome [14]. After the degradation of the proteins, the broken-down cargo is available for recycling.

### 2.2. Mitophagy

There are also selective forms of autophagy that target specific cellular components, such as particular proteins or intracellular organelles. These selective forms of autophagy rely on receptor proteins that determine target-specificity [15]. The first selective process of autophagy to be discussed is mitophagy, which removes the damaged mitochondria for degradation. This process has been implicated in the pathophysiology of neurodegenerative diseases and in maintaining critical physiologic functions, such as the differentiation of red blood cells (RBCs). Mitophagy serves as a quality control mechanism for mitochondria in various tissues and as a process to remove mitochondrial contents. Interest in mitophagy has rapidly grown due to various proteins, such as Parkin and PINK1, which are involved in Alzheimer’s and Parkinson’s diseases [16]. Additional proteins, e.g., BNIP3, FUNDC1, and MURF1, have also been identified as receptors that mediate mitophagy in both physiological and stressed conditions [17]. A greater understanding of this process would reveal vital information regarding the deleterious nature of terminal disease progression.

### 2.3. Reticulophagy

Like mitophagy, reticulophagy leads to the selective degradation of the endoplasmic reticulum (ER) upon ER stress, nutrient deprivation, and nitrogen deficiency [18]. Four ER-resident proteins, FAM134B, RTN3, SEC62, and CCPG1, have been identified as selective autophagy receptors in mammals. Viruses have evolved strategies to destroy FAM134B, avoiding reticulophagy and escaping elimination [19]. Reticulophagy has been involved in many illnesses caused by the Ebola, Dengue, and Zika viruses. Given the diverse functions of the ER and its complexity, additional proteins may be involved in reticulophagy. Studying this process in detail will help to determine potential points of intervention to treat these harmful viral infections.

### 2.4. Microautophagy

Unlike macroautophagy, which utilizes an autophagophore to contain the cytoplasm-derived proteins for degradation, microautophagy involves directly internalizing cytosolic contents into lysosomal vacuoles by engulfing the invaginations of the lysosomal membrane [20]. Microautophagy can selectively uptake either whole organelles or soluble components in the cytosol. The process is upregulated under conditions of cellular stress, such as nutrient deprivation, but studies of this pathway have been limited to yeasts and cell-free systems. Such studies have described four stages of microautophagy: stages I–III comprise the invagination of the lysosomal membrane into its lumen, while stage IV completes the process of uptake by fusing the membrane of the budding vesicle. The four stages are distinguished by their sensitivities to various inhibitors and temperatures. For example, stage I is characterized by its sensitivity to GTPγS, aristolochic acid, and nystatin. Stage II is characterized by its sensitivity to cooling. Stage III is characterized by its sensitivity to FCCP/valinomycin. Lastly, stage IV is characterized by its sensitivity to K252a and rapamycin. Nystatin and a temperature of 0 °C directly interfere with phospholipid membrane invagination and scission, while valinomycin and rapamycin disrupt the membrane electrochemical gradient and cell proliferation, respectively [21,22].

### 2.5. Lipophagy

This is another way by which autophagy contributes to cellular energy stores. The contribution of autophagy to cellular energy was thought to only come from free amino acids (FAAs). However, autophagy can also provide energy through free fatty acids (FFAs). Lipid droplets (LDs) are dynamic and not inert stores, leading to their classification as an intracellular organelle [23,24]. One study showed that when hepatocytes were knocked down for Atg5 and then subjected to an acute oleic acid challenge, there was an overall increase in the size and number of LDs [25]. As was established through biochemical analysis, defective macroautophagy was the sole explanation for this phenomenon [25]. However, lipophagy initiation is poorly understood. The aggregation of ubiquitinated proteins along the surface of LDs, in the presence of proteosomes, has been observed in Atg7-deficient mice [26,27]. These aggregates were eliminated by autophagy, suggesting the targeted activation of lipophagy [28]. Defective lipophagy is characteristic of a variety of pathologies, such as obesity, steatosis, and metabolic syndrome in aging. Animals exposed to high-fat diets for extended periods of time showed an increase in autophagy for the first two weeks, followed by a gradual decrease in autophagic activity. Therefore, this decrease in autophagy predisposes cells to an expansion of the LD compartment, potentially causing hepatotoxicity and steatosis [28-30]. Studies have shown that the pharmacological upregulation of autophagy reduced hepatotoxicity in a fatty liver disease model [31]. Decreased autophagy has been associated with increased age (65) and with decreased lipophagy, which would contribute to an overall increase in LD content; this would serve to further reduce autophagy and cause a vicious circle for the development of hypercholesterolemia and lipid deposits in organs [28]. Lipophagy also has a positive effect on Flavivirus production through unmodified AUP1 [32]. Further studies are needed to characterize how lipophagy is initiated and regulated in both normal and diseased states.

### 2.6. Virophagy (Viral Xenophagy)

Although not entirely understood, virophagy protects against viral infection by linking viral subunits to autophagosomes [33]. Virophagy entails the engulfment of the entire virion, which has been reported in the case of SINV [34]. Additionally, this mechanism could be relevant in anti-viral action against other viruses and warrants further study. Another factor that is thought to be critical in virophagy is the type of cell involved [35]. The contribution of cell type to autophagy is another aspect that needs to be better understood. It is known that this mechanism exists in most cells, but the importance of the activation of this phenomenon in certain cell types over others, and its role in anti-viral activity, could improve our understanding of inflammatory response and innate immunity.

## 3. Autophagy as an Anti-Viral Immune Response

Autophagy is often considered to be an innate immune response, one that is used to control the early stages of viral infection and form a bridge between innate and adaptive responses in which the degradative process is required for antigen processing [36]. Virophagy is an autophagic process that directly degrades intact virions or specific viral proteins. It is triggered by various mechanisms, depending on the virus at hand, some of which will be briefly discussed in Table 1.

For example, upon Sindbis virus (SINV) infection, p62 binds to the viral capsid to activate virophagy [39]. The p62-mediated clearance of SINV in neurons is a host anti-viral response, and the absence of autophagy results in increased neurovirulence. In a hepatitis C virus (HCV) infection, a host ER protein, SHISA5, interacts with the viral nonstructural protein, NS5A, to transport it into the autophagosomes [37]. Vesicular stomatitis virus (VSV) infection triggers a decrease in the cellular PI3K-Akt signaling pathway, which increases autophagy and limits viral replication at the initiation and nucleation stages [40]. During poliovirus infection, the release of viral genome galactosides into the cytoplasm triggers Galectin 8, which initiates autophagy. The initiation step of autophagy, especially, exhibits an anti-viral effect against human immunodeficiency virus-1 (HIV-1), in which the cellular protein, BST2, restricts the viral genome by anchoring it to the cell membrane [41]. Upon norovirus infection, IFN-ɣ induction and the upregulation of GTPases result in the recruitment of the Atg5-Atg12-Atg16L1 complex, which restricts viral replication complexes through a non-degradative mechanism [38]. Plasmacytoid dendritic cells (pDCs) can also detect the presence of a virus without being infected themselves by recognizing viral genomes within lysosomes that have undergone autophagy. When autophagy is inhibited in these cells, interferon (IFN)-α production is also inhibited in response to a range of viruses, such as VSV, Sendai virus (SeV), Influenza virus, HIV-1, and Herpes simplex virus (HSV). This indicates that autophagy is a key process in sensing viruses and initiating an anti-viral response [42].

Autophagy, in addition to its direct anti-viral properties, also plays a role in antigen presentation. Specifically, the autophagy pathway delivers antigens to MHC class I and II molecules, bridging innate and adaptive immune responses. Antigen-presenting cells (APCs) use the transporter associated with antigen processing (TAP) to internalize proteolyzed endogenous antigens, whether viral, tumor, or self, into ER phagosomes containing MHC-I, for presentation to CD8+ T cells. However, studies also implicate autophagy as another mechanism for exogenous antigen internalization and cross-presentation by MHC-I. Treating melanoma cells with specific inhibitors of macroautophagy reduced the MHC-I cross-presentation of the tumor antigen gp100 to corresponding CD8+ cells in vivo [43]. In cases of HIV-1, HSV, or human cytomegalovirus (HCMV) infection, the viral proteins Gag, Gb, and UL138, respectively, are presented through MHC-I and are likely aided by autophagy. Likewise, knocking down Atg12 in EBV-infected cells results in a hampered MHC-II uptake of the viral protein antigen EBNA1, thus blocking T-cell activation. Autophagy also appears to be involved in MHC-II-dependent antigen presentation, even in the absence of viral infection [14]. The induction of autophagy by nutrient deprivation alters the types of peptides expressed on MHC-II, such that the displayed peptides are increasingly derived from intracellular or lysosomal proteins, as opposed to membrane proteins. The increased occurrence of autophagy in APCs, in turn, alters specificity in the subsequent activation of T cells [43].

## 4. Autophagy and the Interferon System

**Figure 3.**
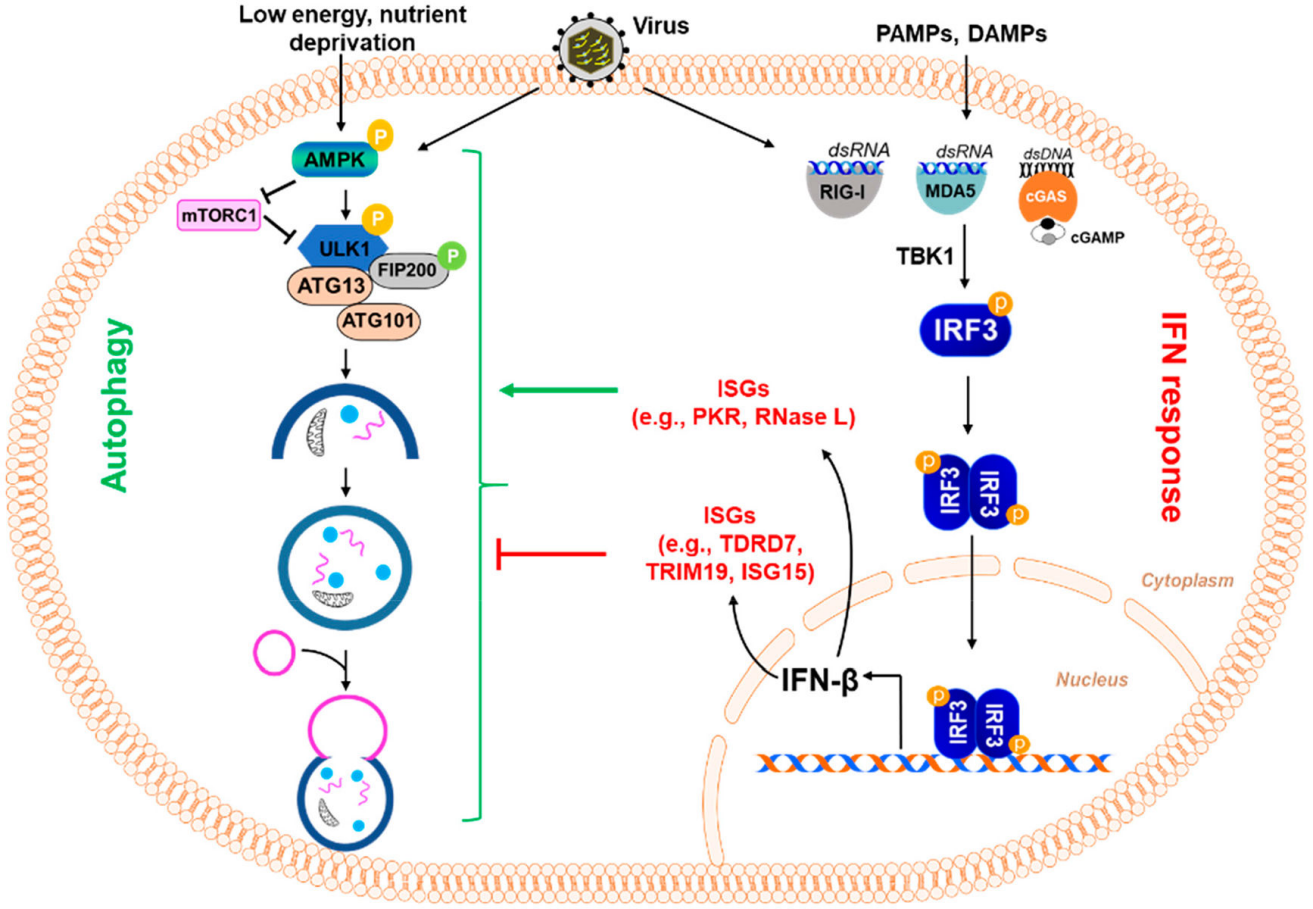
Regulation of the autophagy pathway by interferon-stimulated genes. Virus infection and exposure to PAMPs and DAMPs result in the activation of IRF3, which causes the induction of type-I interferon (e.g., IFNβ) and other IFN-stimulated genes (ISGs) (e.g., TDRD7, TRIM19, ISG15, etc.). These ISGs can inhibit viral replication in different ways, including their ability to inhibit autophagy, which some viruses induce for their own benefit. In this figure, some ISGs are shown because of their ability to inhibit autophagy. TDRD7 inhibits autophagy by blocking the activation of AMPK, the kinase that initiates the autophagy pathway. TRIM19 acts by suppressing virus-induced LC3-I to LC3-II conversion, which is essential for autophagy. Another ISG, ISG15, ISGylates BECN1, critical for autophagy, resulting in its loss of activity and thus inhibiting the induction of autophagy. In contrast, other ISGs, e.g., PKR, RNase L, can activate the autophagy pathway.

Virus infection triggers a robust host immune response, and the interferon (IFN) system is the first line of defense against a wide range of viral infections [44]. Virus-infected cells rapidly induce the production of IFNs, which are secreted and act on neighboring cells via the JAK-STAT signaling pathway. The IFN-induced proteins, also known as IFN-stimulated genes (ISGs), execute the anti-viral action of IFN. ISGs, in addition to directly interfering with the viral life cycle, also regulate the cellular autophagy pathway to control viral replication (Figure 3). The IFN-induced protein kinase R (PKR) inhibits HSV-1 replication in neurons by inducing autophagy; the viral protein ICP34.5 evades PKR-induced autophagy to facilitate replication [45]. PKR is widely known for phosphorylating elf2α to inhibit viral protein translation and form intracellular stress granules that enhance anti-viral signaling. RNase L, another anti-viral ISG known to cleave viral and cellular RNA, induces autophagy by mediating the role of PKR and amplifies the production of IFN [46,47].

Viperin, the virus-inhibitory protein, which is endoplasmic reticulum-associated and interferon-inducible, inhibits the replication of many viruses, such as flaviviruses. The amphipathic nature of viperin allows it to interact with the ER and lipids, diversifying its anti-viral effects [48]. Keeping this in mind, HCV replicates its RNA on lipid droplets by interacting with NS5A, a similar viral amphipathic protein. Further along the autophagy pathway against HCV, viral NS5A is bound by SCOTIN/SHIAS5, another host ISG localized in the ER, leading to its autophagosomal degradation and the inhibition of HCV RNA replication [37]. Both points are proposed mechanistic targets of the anti-HCV action of viperin. Although viperin has been implicated as a therapeutic target against many other viruses, such as DENV, SINV, influenza A, SeV, VSV, HCMV, WNV, and HIV-1, whether there is a unified mechanism for its anti-viral action against all these viruses is not known [48,49]. While we have discussed various ISGs triggered by the presence of viral genomes, there are also IFN-related proteins that are generated by inflammation in response to viruses. As modeled by the *Drosophila* adult brain, the Zika virus (ZIKV) infection of neurons is modulated by an inflammation-induced protein called a *Drosophila* stimulator of IFN genes (dSTING). ZIKV, a flavivirus, initially triggers an NF-kB-dependent inflammatory cascade in the brain, leading to the production of dSTING, which subsequently stimulates IFN-regulatory transcription factor 3 (IRF3) and type I IFN production to induce autophagy and attenuate ZIKV infection in the brain [50].

Another family of immune modulators, known as tripartite motif (TRIM) proteins, often induced by IFN, are ubiquitin E3 ligases that carry out diverse anti-viral functions by either directly inhibiting a viral life cycle or by helping to induce the anti-viral autophagy of virus-infected cells. For instance, TRIM5α interacts with the HIV-1 capsid protein to induce the disassembly of HIV-1 progeny. TRIM proteins play various roles in autophagy induction by upregulating IFN transcription and regulating pattern-recognition receptors with anti-viral signaling cascades. HSV-1, SINV, and adenovirus activate TRIM23’s E3 enzymatic activity, to ubiquitinate cellular proteins linked to viral cargo for their eventual degradation within autophagosomes. TRIM23 is unique in that it also serves as a GTPase to regulate TBK1’s phosphorylation of p62, to enhance cargo recognition in autophagy [51].

In contrast to the various ISGs that induce autophagy to fight against viruses, we have recently discovered an anti-viral ISG, TDRD7, using a high-throughput screen from a library of ISGs. We showed that TDRD7 inhibits virus-induced autophagy to block paramyxovirus replication [6]. Since SeV, RSV, and HPIV3 utilize AMPK, the initiator of the autophagy pathway, we asked whether TDRD7 interferes with AMPK activity. Indeed, our results showed that TDRD7 inhibits AMPK activity in terms of its anti-autophagic and anti-viral functions (Figure 3). We further demonstrated that TDRD7 inhibits HSV-1 replication by its anti-AMPK activity [52]. HSV-1 replication is dependent on AMPK but not the autophagy pathway, and TDRD7 takes advantage of this to inhibit viral replication. We further showed that the TDRD7 anti-viral function is dependent on its ability to inhibit AMPK. The anti-viral function of TDRD7 is diminished in the AMPK inhibitor-treated cells.

Like TDRD7, promyelocytic leukemia (PML), also known as TRIM19, is another ISG in mammalian cells that inhibits autophagy by decreasing viral protein expression. TRIM19 forms aggregates into PML nuclear bodies, which also incorporate other cellular anti-viral restriction factors. TRIM19 has been shown to negatively regulate autophagy in response to an enterovirus 71 (EV71) infection of HeLa cells, as shown by a decrease in the number of autophagosomes and a decrease in the LC3-II/LC3-I ratio when compared to TRIM19 knockout cells [53].

**Figure 4.**
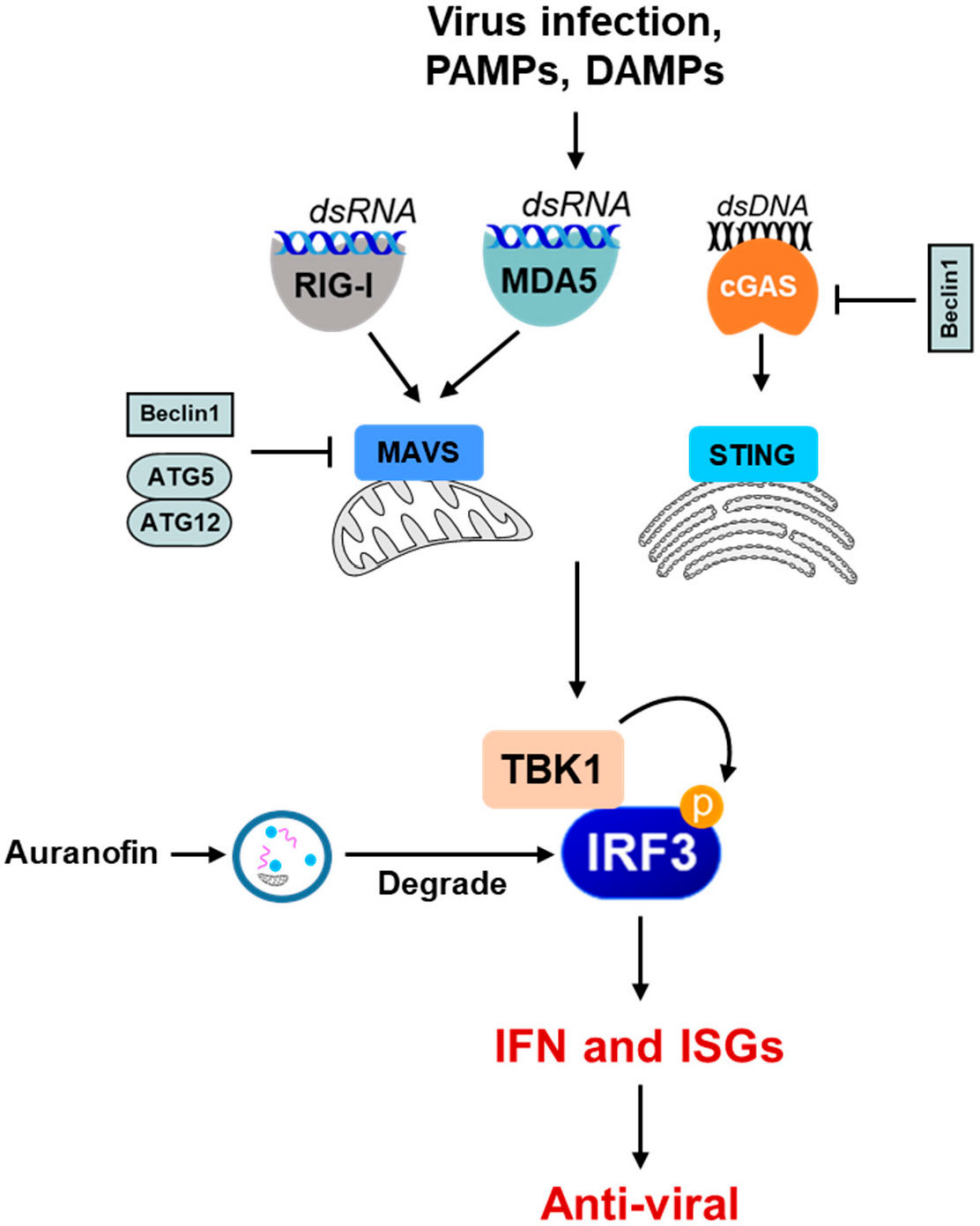
The role of autophagy in the negative regulation of the innate immune signaling pathway. Some examples show that the autophagy machinery or its components inhibit innate immune-sensing pathways (e.g., RIG-I, MDA5, cGAS-STING) in various ways. Beclin1 interacts with MAVS and interferes with the RIG-I signaling pathway, thereby downregulating IFN production. Beclin1 also interacts with cGAS, interfering with the cGAMP synthesis and downstream stimulation of IFN production. The ATG5–ATG12 complex binds to RIG-I CARD and MAVS CARD and negatively regulates IFN production. Selective autophagy also directly degrades the PRRs, such as cGAS, and the adaptor proteins critical in the IFN-I production signaling pathways, such as MAVS, and thus inhibits IFN-I production. Small molecules, e.g., auranofin, can cause the autophagic degradation of the IRF3 protein to inhibit IRF3 activation and IFN induction.

Autophagy-dependent degradation mechanisms can be harmful to IFN-mediated cellular responses. We have recently conducted a high-throughput screen to isolate novel small molecules that regulate viral apoptosis [54]. The screen also isolated auranofin, which inhibited the cellular IFN response [55]. We showed that auranofin activates the cellular autophagy pathway, degrading the IRF3 protein to block the IFN response (Figure 4). The degradation of IRF3 not only inhibits the transcriptional function but also the pro-apoptotic function of IRF3. Therefore, autophagy degrades viral components, as well as causing the degradation of IFN-regulatory proteins [56-59]. The cytoplasmic DNA-sensing pathway by cGAS/STING depends on the cellular autophagy pathway [60-62]. TBK1, a kinase critical for inducing IFN, can also act in the autophagy pathway [63,64], thereby regulating the IFN and autophagy arms of the innate response. Some autophagy pathway proteins can antagonize the innate immune signaling pathways to further regulate the interferon response (Figure 4). Therefore, crosstalk between the IFN and autophagy pathways can be a key regulator of the host response.

## 5. Autophagy as a Pro-Viral Cellular Response

Several viruses often exploit autophagy to facilitate virus replication. The viruses use either a specific structure or a component of the autophagy machinery to promote their replication. HCV uses autophagy in several steps of the viral life cycle; HCV infection activates cellular autophagy, and the inhibition of this process results in decreased viral production. While autophagy inhibition does not affect viral entry, it is necessary to replicate viral RNA and translation [65]. HCV generates autophagosomes, using caveolin 1, caveolin 2, and annexin A2 to generate energy for its replication and promote virion assembly. Additionally, it uses the exocytosis pathway to release progeny virions [36]. As demonstrated, HCV serves as an excellent model of autophagy as a pro-viral mechanism, but countless other viruses similarly utilize parts of the process, many of which are mentioned in this review (Table 2).

Picornaviruses facilitate the formation of autophagosomes to facilitate viral replication, but they block the binding of lysosomes to these autophagosomes. During poliovirus infection, the use of rapamycin, an activator of autophagy, results in increased viral replication. Encephalomyocarditis virus (EMCV) uses its non-structural proteins 2C and 3D to generate ER stress, inducing autophagy and promoting its replication [70]. In Coxsackievirus B3 (CVB3) infection, the inhibition of autophagy by the deletion of Atg5 results in the inhibition of virus replication, specifically in pancreatic acinar cells [73]. We have shown that the Sendai virus (SeV), a paramyxovirus, also depends on cellular autophagy for viral replication [6]. The pharmacological or genetic inhibition of autophagy blocks SeV replication in vitro. SeV uses AMPK, the kinase required to initiate the autophagy pathway, to facilitate virus replication. The inhibition of AMPK by chemicals or genetic approaches blocks SeV replication. We have also shown that AMPK inhibition attenuates human parainfluenza virus (HPIV3) and respiratory syncytial virus (RSV) replication. The inhibition of autophagy and AMPK also attenuate RSV replication and pathogenesis in mice [78]. HPIV3 also triggers mitophagy to inhibit cellular anti-viral responses to IFN, thus advancing its replication [66,67]. During measles virus (MeV) infection, autophagy prevents host-cell death, thereby activating pro-survival pathways that favor viral replication [68]. Coronaviruses also use this autophagic membrane remodeling mechanism to generate viral membranes, as a crucial step in their life cycle [36]. Dengue virus (DENV) induces autophagy by the activation of AMPK, ultimately inducing the formation of a double-membrane vesicle that encloses lipid droplets. This form of autophagy is called lipophagy, which leads to the degradation of lipid droplets via β-oxidation to generate ATP for viral replication [36,79]. Lipophagy, a subset of autophagy where lipid droplets are mobilized within membraned vesicles, serves as a source of energy during infection with flaviviruses such as DENV and WNV. DENV infection leads to the induction of several innate immune responses. However, Dengue virus evades recognition by the host PRRs by replicating within the endoplasmic reticulum, while still using lipids through autophagy and delaying apoptosis to prevent its clearance [71].

The role of autophagy in herpesvirus replication is complex. Varicella-Zoster virus (VZV) has been shown to initiate autophagy upon infection; the chemical inhibition of autophagy or knocking down ATG5 results in decreased viral titer and viral proteins. Autophagy is thus required for the VZV life cycle, and this virus-induced autophagy results in enhanced viral protein synthesis [76]. In contrast to other viruses, HSV-2 does not induce autophagy but instead uses a basal level of autophagy for its own benefit [75]. The inhibition of autophagy in the setting of HSV-2 infection has been shown to hamper viral replication; however, the process is not needed for viral entry into cells. The formation of a double-membrane autophagosome may serve as a protected environment for the generation of progeny virions. HSV-1 replication does not depend on the autophagy pathway. We showed that HSV-1 replication remains unaltered in ATG5-knockdown cells [52]. However, the chemical or genetic inhibition of AMPK attenuates HSV-1 replication. AMPK inhibition in ATG5 knockdown cells also blocks HSV-1 replication. In murine gammaherpesvirus 68 (MHV68) infection, autophagy supports viral reactivation within macrophages. The induction of systemic inflammation, mediated by macrophagic IFN-ɣ, results in the suppression of autophagy genes. Thus, specifically in myeloid cells, Atg genes not only support MHV68 reactivation but also dampen systemic inflammation to curb autophagy inhibition [80].

## 6. Viral Antagonism to Autophagy

While autophagy is an effective mechanism against viral infections, many viruses have evolved to utilize counter-mechanisms that antagonize this anti-viral cellular response. Influenza A virus (IAV) evades cellular autophagy by using its matrix 2 (M2) proteins [74]. The cytoplasmic tail of viral M2 possesses an interaction motif—the LC3 interaction region (LIR)—through which the protein binds to LC3. This binding causes LC3 to re-localize to the plasma membrane, stabilizing influenza virions and facilitating the filamentous budding of the virion. IAV uses a protein–protein interaction to both inhibit anti-viral autophagy and promote its replication [74]. HSV-1 inhibits Beclin-1, a component of the autophagy machinery, to block autophagy induction. The HSV-1 protein ICP34.5 contains a Beclin-1-binding-domain (BBD), which is critical for inhibiting autophagy. The inhibition of autophagy by ICP34.5 results in the development of HSV-1 encephalitis [45,81]. HCMV has two ICP34.5 homologs called TRS1 and IRS1, both of which have BBDs to inhibit cellular autophagy and evade the anti-viral process [82,83]. Kaposi sarcoma-associated herpesvirus (KSHV) possesses a viral Bcl2 analog called ORF16 that mimics cellular Bcl2 and binds Beclin-1, as one of many immune evasion strategies [84]. Although not a member of the Herpesviridae, HIV has also been shown to use Beclin-1 inhibition as a viral countermechanism to autophagy. Several of these viral evasion mechanisms are listed in Table 3.

### Viral Antagonism by SARS-CoV-2

It would be remiss to discuss the viral antagonism of host-cell defense mechanisms without addressing SARS-CoV-2, the beta-coronavirus responsible for the COVID-19 pandemic. Although much of the exact mechanism by which SARS-CoV-2 affects the autophagic pathway is yet to be discovered, AMPK has been shown to be a primary target. Upon the infection of a host cell by the (+)-sense RNA virus, levels of active phosphorylated AMPK (pAMPK) are reduced. Consequently, there is also a decreased presence of the active form of downstream target ULK (pULK), suggesting the inhibition of autophagy initiation as a key mechanism aiding in the CoV-2 evasion of anti-viral defense [90]. Furthermore, accessory factor ORF3a, encoded by the CoV-2 genome, has been identified as a key antagonist of host-cell autophagy. By binding to VPS39, a crucial facilitator of vesicle fusion, ORF3a inhibits the recruitment of Rab7 and the subsequent assembly of the SNARE complex, preventing autophagosome fusion with the lysosome [89]. This multifaceted inhibition of autophagy by the SARS-CoV-2 virus may explain its persistence upon infection.

## 7. Therapeutic Application of Autophagy in Virus Infection

The autophagy pathway can be targeted for therapeutic applications in many diseases, including viral infection [92]. However, because autophagy plays a dual role in viral infection and the immune response, it might be complex to target the autophagy pathway for anti-viral therapy. For example, chloroquine and hydroxychloroquine, originally approved as COVID-19 treatments, are inhibitors of the autophagy pathway [93]. Numerous reports suggest that SARS-CoV-2 entry may depend on the autophagy pathway; therefore, these autophagy inhibitors may be used to counteract the replication of the CoV-2 virus. We have shown that auranofin, an anti-rheumatic compound, induces autophagy to degrade IRF3 [55]. Auranofin has also been shown to inhibit SARS-CoV-2 replication [94]; whether auranofin degrades a viral protein or a pro-viral host protein in this context will require future evaluation. Oncolytic viruses, which are often used in anti-cancer therapy, may function by inducing the autophagy pathway [95]. Anti-retroviral drugs have been shown to impact the cellular autophagy pathway [96]. For example, it was shown that autophagy-promoting drugs, such as everolimus (chemotherapy), carbamazepine (anticonvulsant), and rapamycin (immunotherapy) restricted HIV-1 replication in a cell-specific manner [97]. This highlights the importance of developing and testing new drugs that act on autophagy and re-evaluating previously approved drugs that could be repurposed for therapeutic intervention. We recently characterized a screening strategy that would be useful in repurposing FDA-approved small compounds [54]. Virus-specific therapeutics that impact the autophagy pathway will require in-depth investigation before becoming viable treatment options.

## 8. Conclusions and Future Directions

Autophagy has been studied extensively in virus infection; however, it is not entirely clear how the viruses specifically utilize or are inhibited by autophagy. Similarly, how autophagy regulates the cellular responses to virus infection is under-studied. Since many autophagy-related proteins have roles in regulating ubiquitination, it would be interesting to assess them in autophagy-independent cellular functions. For example, the Atg5-Atg12 complex is involved in RLR signaling, independent of autophagy function [98]. Since viruses have co-evolved along with inhibiting various steps of autophagy, it is considered an anti-viral response of the host. However, viruses may block parts of the autophagy machinery while exploiting other proteins involved in autophagy for their own benefit. Virophagy, the engulfment of the entire virion, although reported in the case of SINV, is not entirely understood. This mechanism could be relevant in anti-viral action against other viruses as well and needs to be studied further. Another factor that is thought to be critical in virophagy is the type of cell involved [99]. The contribution of cell-specific autophagy is, thus, another aspect that needs to be better understood. It is known that this mechanism exists in most cells, but the importance of the activation of this phenomenon in certain cell types over others, and its role in anti-viral activity, could improve our understanding of innate immunity. Several autophagic proteins have been found to interact with viral proteins, ranging across five RNA virus families, as part of an interactome study [100]. These have yet to be studied in terms of a direct role in the restriction of viruses.

## Figures and Tables

**Table 1.** Anti-viral action of autophagy against specific viruses.

Virus Inhibited	Autophagy Step	Mechanism	References
Hepatitis C Virus (HCV)	Elongation	SCOTIN associates with viral NS5A, leading to its degradation	[37]
Poliovirus	Cargo Selection	Galectin 8 marks permeated endosomes for autophagic destruction of the viral genome	[10]
Norovirus (NoV)	Nucleation	IFN-ɣ and GTPases recruit Atg5-Atg12-Atg16L1 complex to restrict the virus	[38]
Sindbis Virus (SINV)	Cargo Selection	p62 binds to viral capsid protein to target the virus to autophagosome	[39]
Vesicular Stomatitis Virus (VSV)	Initiation and Nucleation	VSV-G surface glycoprotein initiates the anti-viral autophagic pathway controlled by PI3K/Akt	[40]
Herpes Simplex Virus (HSV)	Cargo Selection	An LC3 like protein is derived from the viral nucleus to bind to the autophagosome	[36]
Human Immunodeficiency Virus (HIV-1)	Initiation	BST2 ectodomain anchors the HIV genome to cell membranes, to restrict virion release	[41]

**Table 2.** Virus-induced autophagy as a pro-viral response.

Virus	Viral Protein	Mechanism	References
Human Parainfluenza Virus 3 (HPIV3)		Induces autophagy through AMPK for replication	[6,66,67]
Measles Virus (MeV)	MeV-C protein	MeV binds CD46 to induce initial autophagy C protein binds host IRGM to induce a second autophagy wave after viral replication, to prevent cell death	[68,69]
Sendai Virus (SeV)		Induces autophagy through AMPK for replication	[6]
Encephalomyocarditis Virus (EMCV)	Non-structural proteins 2C and 3D (NS2C/3D)	Induces autophagy through the ER stress pathway	[70]
Dengue Virus (DENV)	NS2B/3	Induces ER stress through XBP1 and lipophagy to use the resulting ATP	[36,71]
West Nile Virus (WNV)	NS2B/3	Induces lipophagy to use the resulting ATP	[36]
Hepatitis C Virus (HCV)	NS3/4A	Interacts with host annexin-A2 to use autophagosomal lipid rafts during viral RNA translation	[36,72]
Coxsackievirus B		Induces autophagy, to use the membrane for viral RNA replication	[73]
Influenza A Virus (IAV)	M2 protein LC3-interacting region	M2 protein interacts with LC3 to relocate the virus to the plasma membrane for budding	[74]
Herpes Simplex Virus-1 (HSV-1)	TAP-blocking protein	Promotes use of autophagosome for protection during antigen presentation	[36]
Herpes Simplex Virus-2 (HSV-2)		Maintains basal level of autophagy for infection	[75]
Varicella Zoster Virus (VZV)		Induction of autophagy for viral glycoprotein synthesis	[76]
Human Immunodeficiency Virus (HIV-1)	Viral protein u (Vpu)	Removes BST2 from the viral budding sites to allow the spread of new virions	[77]

**Table 3.** Viral antagonism to autophagy.

Virus	Viral Protein	Mechanism	References
Poliovirus	2BC, 3A	Induces LC3 lipidation and double-membraned vesicle formation for replication	[36,85]
	HRAS-like suppressor 3 (PLA2G16)	Escapes autophagic degradation by evading detection of its genome-containing endosomes	[36,86]
Hepatitis C Virus (HCV)	RdRP NS5B	Binds Atg5	[36]
	NS4B	Induces UVRAG and Rubicon to enhance autophagic flux temporarily	
	Unknown	Targets host IRGM to fragment the Golgi apparatus	
Foot-and-Mouth Disease Virus (FMDV)	VP1 capsid protein	Associates with p62 to use autophagosomes after the initial induction of autophagy	[36,87]
Zika Virus (ZIKV)	NS4A/4B	Inhibit AKT phosphorylation and mTOR activation	[36,88]
Coronavirus (SARS-CoV-2)	ORF3a	Binds to VPS39 and inhibits recruitment of Rab7 and the subsequent assembly of the SNARE complex, preventing autophagosome fusion with the lysosome	[89,90]
Human Simplex Virus 1 (HSV-1)	ICP34.5	Inhibits Beclin 1	[36]
Human Cytomegalovirus (HCMV)	TRS1, IRS1	Inhibits Beclin 1	[36,82]
Kaposi’s Sarcoma-associated Herpesvirus (KSHV)	vBcl-2	Inhibits Beclin 1	[84]
	vFLIP	Blocks Atg3 E2 enzyme and the lipidation of LC3	
Human Immuno-deficiency Virus (HIV-1)	Nef	Inhibits Beclin 1	[36,91]

## References

[1] Kawai T; Akira S Innate immune recognition of viral infection. Nat. Immunol 2006, 7, 131–137. 16424890 10.1038/ni1303

[2] Thompson MR; Kaminski JJ; Kurt-Jones EA; Fitzgerald KA Pattern recognition receptors and the innate immune response to viral infection. Viruses 2011, 3, 920–940. 21994762 10.3390/v3060920 PMC3186011

[3] Carty M; Guy C; Bowie AG Detection of Viral Infections by Innate Immunity. Biochem. Pharmacol 2021, 183, 114316. 33152343 10.1016/j.bcp.2020.114316

[4] Iwasaki A; Medzhitov R Control of adaptive immunity by the innate immune system. Nat. Immunol 2015, 16, 343–353. 25789684 10.1038/ni.3123 PMC4507498

[5] Guidotti LG; Chisari FV Noncytolytic control of viral infections by the innate and adaptive immune response. Annu. Rev. Immunol 2001, 19, 65–91. 11244031 10.1146/annurev.immunol.19.1.65

[6] Subramanian G; Kuzmanovic T; Zhang Y; Peter CB; Veleeparambil M; Chakravarti R; Sen GC; Chattopadhyay S A new mechanism of interferon’s antiviral action: Induction of autophagy, essential for paramyxovirus replication, is inhibited by the interferon stimulated gene, TDRD7. PLoS Pathog. 2018, 14, e1006877. 29381763 10.1371/journal.ppat.1006877 PMC5806901

[7] Jackson WT Viruses and the autophagy pathway. Virology 2015, 479–480, 450–456. 10.1016/j.virol.2015.03.042 PMC5917100 25858140

[8] Ichimiya T; Yamakawa T; Hirano T; Yokoyama Y; Hayashi Y; Hirayama D; Wagatsuma K; Itoi T; Nakase H Autophagy and Autophagy-Related Diseases: A Review. Int. J. Mol. Sci 2020, 21, 8974. 10.3390/ijms21238974 PMC7729615 33255983

[9] Ahmad L; Mostowy S; Sancho-Shimizu V Autophagy-Virus Interplay: From Cell Biology to Human Disease. Front. Cell Dev. Biol 2018, 6, 155. 30510929 10.3389/fcell.2018.00155 PMC6252315

[10] Mao J; Lin E; He L; Yu J; Tan P; Zhou Y Autophagy and Viral Infection. Adv. Exp. Med. Biol 2019, 1209, 55–78. 31728865 10.1007/978-981-15-0606-2_5 PMC7122562

[11] Levy JMM; Towers CG; Thorburn A Targeting autophagy in cancer. Nat. Rev. Cancer 2017, 17, 528–542. 28751651 10.1038/nrc.2017.53 PMC5975367

[12] Pereira G; Leao A; Erustes AG; Morais IBM; Vrechi TAM; Zamarioli LDS; Pereira CAS; Marchioro LO; Sperandio LP; Lins IVF; Pharmacological Modulators of Autophagy as a Potential Strategy for the Treatment of COVID-19. Int. J. Mol. Sci 2021, 22, 4067. 33920748 10.3390/ijms22084067 PMC8071111

[13] Dikic I; Elazar Z Mechanism and medical implications of mammalian autophagy. Nat. Rev. Mol. Cell Biol 2018, 19, 349–364. 29618831 10.1038/s41580-018-0003-4

[14] Chiramel AI; Brady NR; Bartenschlager R Divergent roles of autophagy in virus infection. Cells 2013, 2, 83–104. 24709646 10.3390/cells2010083 PMC3972664

[15] Farre JC; Subramani S Mechanistic insights into selective autophagy pathways: Lessons from yeast. Nat. Rev. Mol. Cell Biol 2016, 17, 537–552. 27381245 10.1038/nrm.2016.74 PMC5549613

[16] Quinn PMJ; Moreira PI; Ambrosio AF; Alves CH PINK1/PARKIN signalling in neurodegeneration and neuroinflammation. Acta Neuropathol. Commun 2020, 8, 189. 33168089 10.1186/s40478-020-01062-w PMC7654589

[17] Liu L; Sakakibara K; Chen Q; Okamoto K Receptor-mediated mitophagy in yeast and mammalian systems. Cell Res. 2014, 24, 787–795. 24903109 10.1038/cr.2014.75 PMC4085769

[18] Nakatogawa H; Mochida K Reticulophagy and nucleophagy: New findings and unsolved issues. Autophagy 2015, 11, 2377–2378. 26566146 10.1080/15548627.2015.1106665 PMC4835146

[19] Lennemann NJ; Coyne CB Dengue and Zika viruses subvert reticulophagy by NS2B3-mediated cleavage of FAM134B. Autophagy 2017, 13, 322–332. 28102736 10.1080/15548627.2016.1265192 PMC5324851

[20] Li WW; Li J; Bao JK Microautophagy: Lesser-known self-eating. Cell. Mol. Life Sci 2012, 69, 1125–1136. 22080117 10.1007/s00018-011-0865-5 PMC11114512

[21] Crotzer VL; Blum JS Autophagy and adaptive immunity. Immunology 2010, 131, 9–17. 20586810 10.1111/j.1365-2567.2010.03321.x PMC2966753

[22] Kunz JB; Schwarz H; Mayer A Determination of four sequential stages during microautophagy in vitro. J. Biol. Chem 2004, 279, 9987–9996. 14679207 10.1074/jbc.M307905200

[23] Greenberg AS; Coleman RA; Kraemer FB; McManaman JL; Obin MS; Puri V; Yan QW; Miyoshi H; Mashek DG The role of lipid droplets in metabolic disease in rodents and humans. J. Clin. Investig 2011, 121, 2102–2110. 21633178 10.1172/JCI46069 PMC3104768

[24] Fujimoto T; Parton RG Not just fat: The structure and function of the lipid droplet. Cold Spring Harb. Perspect. Biol 2011, 3, a004838. 21421923 10.1101/cshperspect.a004838 PMC3039932

[25] Singh R; Kaushik S; Wang Y; Xiang Y; Novak I; Komatsu M; Tanaka K; Cuervo AM; Czaja MJ Autophagy regulates lipid metabolism. Nature 2009, 458, 1131–1135. 19339967 10.1038/nature07976 PMC2676208

[26] Komatsu M; Waguri S; Ueno T; Iwata J; Murata S; Tanida I; Ezaki J; Mizushima N; Ohsumi Y; Uchiyama Y; Impairment of starvation-induced and constitutive autophagy in Atg7-deficient mice. J. Cell Biol 2005, 169, 425–434. 15866887 10.1083/jcb.200412022 PMC2171928

[27] Ohsaki Y; Cheng J; Fujita A; Tokumoto T; Fujimoto T Cytoplasmic lipid droplets are sites of convergence of proteasomal and autophagic degradation of apolipoprotein B. Mol. Biol. Cell 2006, 17, 2674–2683. 16597703 10.1091/mbc.E05-07-0659 PMC1474802

[28] Singh R; Cuervo AM Lipophagy: Connecting autophagy and lipid metabolism. Int. J. Cell Biol 2012, 2012, 282041. 22536247 10.1155/2012/282041 PMC3320019

[29] Koga H; Kaushik S; Cuervo AM Altered lipid content inhibits autophagic vesicular fusion. FASEB J. 2010, 24, 3052–3065. 20375270 10.1096/fj.09-144519 PMC2909278

[30] Mei S; Ni HM; Manley S; Bockus A; Kassel KM; Luyendyk JP; Copple BL; Ding WX Differential roles of unsaturated and saturated fatty acids on autophagy and apoptosis in hepatocytes. J. Pharmacol. Exp. Ther 2011, 339, 487–498. 21856859 10.1124/jpet.111.184341 PMC3199993

[31] Thoen LF; Guimaraes EL; Dolle L; Mannaerts I; Najimi M; Sokal E; van Grunsven LA A role for autophagy during hepatic stellate cell activation. J. Hepatol 2011, 55, 1353–1360. 21803012 10.1016/j.jhep.2011.07.010

[32] Zhang J; Lan Y; Li MY; Lamers MM; Fusade-Boyer M; Klemm E; Thiele C; Ashour J; Sanyal S Flaviviruses Exploit the Lipid Droplet Protein AUP1 to Trigger Lipophagy and Drive Virus Production. Cell Host Microbe 2018, 23, 819–831.e5. 29902443 10.1016/j.chom.2018.05.005

[33] Tiwari SK; Dang JW; Lin N; Qin Y; Wang S; Rana TM Zika virus depletes neural stem cells and evades selective autophagy by suppressing the Fanconi anemia protein FANCC. EMBO Rep. 2020, 21, e49183. 33073500 10.15252/embr.201949183 PMC7726779

[34] Sumpter R Jr.; Sirasanagandla S; Fernandez AF; Wei Y; Dong X; Franco L; Zou Z; Marchal C; Lee MY; Clapp DW; Fanconi Anemia Proteins Function in Mitophagy and Immunity. Cell 2016, 165, 867–881. 27133164 10.1016/j.cell.2016.04.006 PMC4881391

[35] Viret C; Rozieres A; Faure M Novel Insights into NDP52 Autophagy Receptor Functioning. Trends Cell Biol. 2018, 28, 255–257. 29395717 10.1016/j.tcb.2018.01.003

[36] Choi Y; Bowman JW; Jung JU Autophagy during viral infection—A double-edged sword. Nat. Rev. Microbiol 2018, 16, 341–354. 29556036 10.1038/s41579-018-0003-6 PMC6907743

[37] Kim N; Kim MJ; Sung PS; Bae YC; Shin EC; Yoo JY Interferon-inducible protein SCOTIN interferes with HCV replication through the autolysosomal degradation of NS5A. Nat. Commun 2016, 7, 10631. 26868272 10.1038/ncomms10631 PMC4754343

[38] Hwang S; Maloney NS; Bruinsma MW; Goel G; Duan E; Zhang L; Shrestha B; Diamond MS; Dani A; Sosnovtsev SV; Nondegradative role of Atg5-Atg12/Atg16L1 autophagy protein complex in antiviral activity of interferon gamma. Cell Host Microbe 2012, 11, 397–409. 22520467 10.1016/j.chom.2012.03.002 PMC3348177

[39] Orvedahl A; MacPherson S; Sumpter R Jr.; Talloczy Z; Zou Z; Levine B Autophagy protects against Sindbis virus infection of the central nervous system. Cell Host Microbe 2010, 7, 115–127. 20159618 10.1016/j.chom.2010.01.007 PMC2860265

[40] Shelly S; Lukinova N; Bambina S; Berman A; Cherry S Autophagy is an essential component of Drosophila immunity against vesicular stomatitis virus. Immunity 2009, 30, 588–598. 19362021 10.1016/j.immuni.2009.02.009 PMC2754303

[41] Tokarev A; Suarez M; Kwan W; Fitzpatrick K; Singh R; Guatelli J Stimulation of NF-kappaB activity by the HIV restriction factor BST2. J. Virol 2013, 87, 2046–2057. 23221546 10.1128/JVI.02272-12 PMC3571454

[42] Lee HK; Lund JM; Ramanathan B; Mizushima N; Iwasaki A Autophagy-dependent viral recognition by plasmacytoid dendritic cells. Science 2007, 315, 1398–1401. 17272685 10.1126/science.1136880

[43] Crotzer VL; Blum JS Autophagy and its role in MHC-mediated antigen presentation. J. Immunol 2009, 182, 3335–3341. 19265109 10.4049/jimmunol.0803458 PMC2730830

[44] Glanz A; Chakravarty S; Varghese M; Kottapalli A; Fan S; Chakravarti R; Chattopadhyay S Transcriptional and Non-Transcriptional Activation, Posttranslational Modifications, and Antiviral Functions of Interferon Regulatory Factor 3 and Viral Antagonism by the SARS-Coronavirus. Viruses 2021, 13, 575. 33805458 10.3390/v13040575 PMC8066409

[45] Orvedahl A; Alexander D; Talloczy Z; Sun Q; Wei Y; Zhang W; Burns D; Leib DA; Levine B HSV-1 ICP34.5 confers neurovirulence by targeting the Beclin 1 autophagy protein. Cell Host Microbe 2007, 1, 23–35. 18005679 10.1016/j.chom.2006.12.001

[46] Siddiqui MA; Malathi K RNase L induces autophagy via c-Jun N-terminal kinase and double-stranded RNA-dependent protein kinase signaling pathways. J. Biol. Chem 2012, 287, 43651–43664. 23109342 10.1074/jbc.M112.399964 PMC3527951

[47] Manivannan P; Siddiqui MA; Malathi K RNase L Amplifies Interferon Signaling by Inducing Protein Kinase R-Mediated Antiviral Stress Granules. J. Virol 2020, 94, e00205–20. 32295917 10.1128/JVI.00205-20 PMC7307175

[48] Seo JY; Yaneva R; Cresswell P Viperin: A multifunctional, interferon-inducible protein that regulates virus replication. Cell Host Microbe 2011, 10, 534–539. 22177558 10.1016/j.chom.2011.11.004 PMC3246677

[49] Seo JY; Cresswell P Viperin regulates cellular lipid metabolism during human cytomegalovirus infection. PLoS Pathog. 2013, 9, e1003497. 23935494 10.1371/journal.ppat.1003497 PMC3731232

[50] Liu Y; Gordesky-Gold B; Leney-Greene M; Weinbren NL; Tudor M; Cherry S Inflammation-Induced, STING-Dependent Autophagy Restricts Zika Virus Infection in the Drosophila Brain. Cell Host Microbe 2018, 24, 57–68.e3. 29934091 10.1016/j.chom.2018.05.022 PMC6173519

[51] van Gent M; Sparrer KMJ; Gack MU TRIM Proteins and Their Roles in Antiviral Host Defenses. Annu. Rev. Virol 2018, 5, 385–405. 29949725 10.1146/annurev-virology-092917-043323 PMC6186430

[52] Subramanian G; Popli S; Chakravarty S; Taylor RT; Chakravarti R; Chattopadhyay S The interferon-inducible protein TDRD7 inhibits AMP-activated protein kinase and thereby restricts autophagy-independent virus replication. J. Biol. Chem 2020, 295, 6811–6822. 32273341 10.1074/jbc.RA120.013533 PMC7242695

[53] Chen D; Feng C; Tian X; Zheng N; Wu Z Promyelocytic Leukemia Restricts Enterovirus 71 Replication by Inhibiting Autophagy. Front. Immunol 2018, 9, 1268. 29922292 10.3389/fimmu.2018.01268 PMC5996053

[54] Glanz A; Chawla K; Fabry S; Subramanian G; Garcia J; Jay B; Ciricillo J; Chakravarti R; Taylor RT; Chattopadhyay S High Throughput Screening of FDA-Approved Drug Library Reveals the Compounds that Promote IRF3-Mediated Pro-Apoptotic Pathway Inhibit Virus Replication. Viruses 2020, 12, 442. 10.3390/v12040442 PMC7232324 32295140

[55] Glanz A; Chakravarty S; Fan S; Chawla K; Subramanian G; Rahman T; Walters D; Chakravarti R; Chattopadhyay S Autophagic degradation of IRF3 induced by the small-molecule auranofin inhibits its transcriptional and proapoptotic activities. J. Biol. Chem 2021, 297, 101274. 34619149 10.1016/j.jbc.2021.101274 PMC8531670

[56] Xie W; Jin S; Zhang C; Yang S; Wu Y; Zhao Y; Songyang Z; Cui J Selective autophagy controls the stability of TBK1 via NEDD4 to balance host defense. Cell Death Differ. 2021, 29, 40–53. 34257412 10.1038/s41418-021-00833-9 PMC8738727

[57] Liu J; Wu X; Wang H; Wei J; Wu Q; Wang X; Yan Y; Cui J; Min J; Wang F; HFE inhibits type I IFNs signaling by targeting the SQSTM1-mediated MAVS autophagic degradation. Autophagy 2021, 17, 1962–1977. 32746697 10.1080/15548627.2020.1804683 PMC8386699

[58] Wu Y; Jin S; Liu Q; Zhang Y; Ma L; Zhao Z; Yang S; Li YP; Cui J Selective autophagy controls the stability of transcription factor IRF3 to balance type I interferon production and immune suppression. Autophagy 2021, 17, 1379–1392. 32476569 10.1080/15548627.2020.1761653 PMC8205069

[59] Kimura T; Jain A; Choi SW; Mandell MA; Schroder K; Johansen T; Deretic V TRIM-mediated precision autophagy targets cytoplasmic regulators of innate immunity. J. Cell Biol 2015, 210, 973–989. 26347139 10.1083/jcb.201503023 PMC4576868

[60] Gui X; Yang H; Li T; Tan X; Shi P; Li M; Du F; Chen ZJ Autophagy induction via STING trafficking is a primordial function of the cGAS pathway. Nature 2019, 567, 262–266. 30842662 10.1038/s41586-019-1006-9 PMC9417302

[61] Prantner D; Perkins DJ; Vogel SN AMP-activated Kinase (AMPK) Promotes Innate Immunity and Antiviral Defense through Modulation of Stimulator of Interferon Genes (STING) Signaling. J. Biol. Chem 2017, 292, 292–304. 27879319 10.1074/jbc.M116.763268 PMC5217687

[62] Liang Q; Seo GJ; Choi YJ; Ge J; Rodgers MA; Shi M; Jung JU Autophagy side of MB21D1/cGAS DNA sensor. Autophagy 2014, 10, 1146–1147. 24879161 10.4161/auto.28769 PMC4091176

[63] Herhaus L; Bhaskara RM; Lystad AH; Gestal-Mato U; Covarrubias-Pinto A; Bonn F; Simonsen A; Hummer G; Dikic I TBK1-mediated phosphorylation of LC3C and GABARAP-L2 controls autophagosome shedding by ATG4 protease. EMBO Rep. 2020, 21, e48317. 31709703 10.15252/embr.201948317 PMC6945063

[64] Weidberg H; Elazar Z TBK1 mediates crosstalk between the innate immune response and autophagy. Sci. Signal 2011, 4, pe39. 21868362 10.1126/scisignal.2002355

[65] Dreux M; Gastaminza P; Wieland SF; Chisari FV The autophagy machinery is required to initiate hepatitis C virus replication. Proc. Natl. Acad. Sci. USA 2009, 106, 14046–14051. 19666601 10.1073/pnas.0907344106 PMC2729017

[66] Zhang L; Qin Y; Chen M Viral strategies for triggering and manipulating mitophagy. Autophagy 2018, 14, 1665–1673. 29895192 10.1080/15548627.2018.1466014 PMC6135629

[67] Ding B; Zhang L; Li Z; Zhong Y; Tang Q; Qin Y; Chen M The Matrix Protein of Human Parainfluenza Virus Type 3 Induces Mitophagy that Suppresses Interferon Responses. Cell Host Microbe 2017, 21, 538–547.e4. 28407488 10.1016/j.chom.2017.03.004

[68] Lennemann NJ; Coyne CB Catch me if you can: The link between autophagy and viruses. PLoS Pathog. 2015, 11, e1004685. 25811485 10.1371/journal.ppat.1004685 PMC4374752

[69] Richetta C; Gregoire IP; Verlhac P; Azocar O; Baguet J; Flacher M; Tangy F; Rabourdin-Combe C; Faure M Sustained autophagy contributes to measles virus infectivity. PLoS Pathog. 2013, 9, e1003599. 24086130 10.1371/journal.ppat.1003599 PMC3784470

[70] Hou L; Ge X; Xin L; Zhou L; Guo X; Yang H Nonstructural proteins 2C and 3D are involved in autophagy as induced by the encephalomyocarditis virus. Virol. J 2014, 11, 156. 25178311 10.1186/1743-422X-11-156 PMC4161894

[71] Green AM; Beatty PR; Hadjilaou A; Harris E Innate immunity to dengue virus infection and subversion of antiviral responses. J. Mol. Biol 2014, 426, 1148–1160. 24316047 10.1016/j.jmb.2013.11.023 PMC4174300

[72] Saxena V; Lai CK; Chao TC; Jeng KS; Lai MM Annexin A2 is involved in the formation of hepatitis C virus replication complex on the lipid raft. J. Virol 2012, 86, 4139–4150. 22301157 10.1128/JVI.06327-11 PMC3318618

[73] Alirezaei M; Flynn CT; Wood MR; Whitton JL Pancreatic acinar cell-specific autophagy disruption reduces coxsackievirus replication and pathogenesis in vivo. Cell Host Microbe 2012,11, 298–305. 22423969 10.1016/j.chom.2012.01.014 PMC3308121

[74] Beale R; Wise H; Stuart A; Ravenhill BJ; Digard P; Randow F A LC3-interacting motif in the influenza A virus M2 protein is required to subvert autophagy and maintain virion stability. Cell Host Microbe 2014, 15, 239–247. 24528869 10.1016/j.chom.2014.01.006 PMC3991421

[75] Yakoub AM; Shukla D Basal Autophagy Is Required for Herpes simplex Virus-2 Infection. Sci. Rep 2015, 5, 12985. 26248741 10.1038/srep12985 PMC4528227

[76] Buckingham EM; Carpenter JE; Jackson W; Grose C Autophagy and the effects of its inhibition on varicella-zoster virus glycoprotein biosynthesis and infectivity. J. Virol 2014, 88, 890–902. 24198400 10.1128/JVI.02646-13 PMC3911683

[77] Lukhele S; Cohen EA Conserved residues within the HIV-1 Vpu transmembrane-proximal hinge region modulate BST2 binding and antagonism. Retrovirology 2017, 14, 18. 28288652 10.1186/s12977-017-0345-6 PMC5348903

[78] Li M; Li J; Zeng R; Yang J; Liu J; Zhang Z; Song X; Yao Z; Ma C; Li W; Respiratory Syncytial Virus Replication Is Promoted by Autophagy-Mediated Inhibition of Apoptosis. J. Virol 2018, 92, e02193–17. 29386287 10.1128/JVI.02193-17 PMC5874425

[79] Jordan TX; Randall G Dengue Virus Activates the AMP Kinase-mTOR Axis To Stimulate a Proviral Lipophagy. J. Virol 2017, 91, e02020–16. 28298606 10.1128/JVI.02020-16 PMC5432877

[80] Park S; Buck MD; Desai C; Zhang X; Loginicheva E; Martinez J; Freeman ML; Saitoh T; Akira S; Guan JL; Autophagy Genes Enhance Murine Gammaherpesvirus 68 Reactivation from Latency by Preventing Virus-Induced Systemic Inflammation. Cell Host Microbe 2016, 19, 91–101. 26764599 10.1016/j.chom.2015.12.010 PMC4714357

[81] Yordy B; Iijima N; Huttner A; Leib D; Iwasaki A A neuron-specific role for autophagy in antiviral defense against herpes simplex virus. Cell Host Microbe 2012, 12, 334–345. 22980330 10.1016/j.chom.2012.07.013 PMC3454454

[82] Chaumorcel M; Lussignol M; Mouna L; Cavignac Y; Fahie K; Cotte-Laffitte J; Geballe A; Brune W; Beau I; Codogno P; The human cytomegalovirus protein TRS1 inhibits autophagy via its interaction with Beclin 1. J. Virol 2012, 86, 2571–2584. 22205736 10.1128/JVI.05746-11 PMC3302257

[83] Mouna L; Hernandez E; Bonte D; Brost R; Amazit L; Delgui LR; Brune W; Geballe AP; Beau I; Esclatine A Analysis of the role of autophagy inhibition by two complementary human cytomegalovirus BECN1/Beclin 1-binding proteins. Autophagy 2016, 12, 327–342. 26654401 10.1080/15548627.2015.1125071 PMC4836022

[84] Liang Q; Wei D; Chung B; Brulois KF; Guo C; Dong S; Gao SJ; Feng P; Liang C; Jung JU Novel Role of vBcl2 in the Virion Assembly of Kaposi’s Sarcoma-Associated Herpesvirus. J. Virol 2018, 92, e00914–17. 29167347 10.1128/JVI.00914-17 PMC5790944

[85] Jackson WT; Giddings TH Jr.; Taylor MP; Mulinyawe S; Rabinovitch M; Kopito RR; Kirkegaard K Subversion of cellular autophagosomal machinery by RNA viruses. PLoS Biol. 2005, 3, e156. 15884975 10.1371/journal.pbio.0030156 PMC1084330

[86] Staring J; von Castelmur E; Blomen VA; van den Hengel LG; Brockmann M; Baggen J; Thibaut HJ; Nieuwenhuis J; Janssen H; van Kuppeveld FJ; PLA2G16 represents a switch between entry and clearance of Picornaviridae. Nature 2017, 541, 412–416. 28077878 10.1038/nature21032

[87] O’Donnell V; Pacheco JM; LaRocco M; Burrage T; Jackson W; Rodriguez LL; Borca MV; Baxt B Foot-and-mouth disease virus utilizes an autophagic pathway during viral replication. Virology 2011, 410, 142–150. 21112602 10.1016/j.virol.2010.10.042 PMC7126820

[88] Liang Q; Luo Z; Zeng J; Chen W; Foo SS; Lee SA; Ge J; Wang S; Goldman SA; Zlokovic BV; Zika Virus NS4A and NS4B Proteins Deregulate Akt-mTOR Signaling in Human Fetal Neural Stem Cells to Inhibit Neurogenesis and Induce Autophagy. Cell Stem Cell 2016, 19, 663–671. 27524440 10.1016/j.stem.2016.07.019 PMC5144538

[89] Zhang Y; Sun H; Pei R; Mao B; Zhao Z; Li H; Lin Y; Lu K The SARS-CoV-2 protein ORF3a inhibits fusion of autophagosomes with lysosomes. Cell Discov. 2021, 7, 31. 33947832 10.1038/s41421-021-00268-z PMC8096138

[90] Gassen NC; Papies J; Bajaj T; Emanuel J; Dethloff F; Chua RL; Trimpert J; Heinemann N; Niemeyer C; Weege F; SARS-CoV-2-mediated dysregulation of metabolism and autophagy uncovers host-targeting antivirals. Nat. Commun 2021, 12, 3818. 34155207 10.1038/s41467-021-24007-w PMC8217552

[91] Kyei GB; Dinkins C; Davis AS; Roberts E; Singh SB; Dong C; Wu L; Kominami E; Ueno T; Yamamoto A; Autophagy pathway intersects with HIV-1 biosynthesis and regulates viral yields in macrophages. J. Cell Biol 2009, 186, 255–268. 19635843 10.1083/jcb.200903070 PMC2717652

[92] Rubinsztein DC; Codogno P; Levine B Autophagy modulation as a potential therapeutic target for diverse diseases. Nat. Rev. Drug Discov 2012, 11, 709–730. 22935804 10.1038/nrd3802 PMC3518431

[93] Maity S; Saha A Therapeutic Potential of Exploiting Autophagy Cascade Against Coronavirus Infection. Front. Microbiol 2021, 12, 675419. 34054782 10.3389/fmicb.2021.675419 PMC8160449

[94] Rothan HA; Stone S; Natekar J; Kumari P; Arora K; Kumar M The FDA-approved gold drug auranofin inhibits novel coronavirus (SARS-CoV-2) replication and attenuates inflammation in human cells. Virology 2020, 547, 7–11. 32442105 10.1016/j.virol.2020.05.002 PMC7236683

[95] Hu L; Jiang K; Ding C; Meng S Targeting Autophagy for Oncolytic Immunotherapy. Biomedicines 2017, 5, 5. 10.3390/biomedicines5010005 PMC5423490 28536348

[96] Cheney L; Barbaro JM; Berman JW Antiretroviral Drugs Impact Autophagy with Toxic Outcomes. Cells 2021, 10, 909. 33920955 10.3390/cells10040909 PMC8071244

[97] Cloherty APM; van Teijlingen NH; Eisden T; van Hamme JL; Rader AG; Geijtenbeek TBH; Schreurs R; Ribeiro CMS Autophagy-enhancing drugs limit mucosal HIV-1 acquisition and suppress viral replication ex vivo. Sci. Rep 2021, 11, 4767. 33637808 10.1038/s41598-021-84081-4 PMC7910550

[98] Jounai N; Takeshita F; Kobiyama K; Sawano A; Miyawaki A; Xin KQ; Ishii KJ; Kawai T; Akira S; Suzuki K; The Atg5 Atg12 conjugate associates with innate antiviral immune responses. Proc. Natl. Acad. Sci. USA 2007, 104, 14050–14055. 17709747 10.1073/pnas.0704014104 PMC1955809

[99] Viret C; Rozieres A; Faure M Autophagy during Early Virus-Host Cell Interactions. J. Mol. Biol 2018, 430, 1696–1713. 29698649 10.1016/j.jmb.2018.04.018

[100] Gregoire IP; Rabourdin-Combe C; Faure M Autophagy and RNA virus interactomes reveal IRGM as a common target. Autophagy 2012, 8, 1136–1137. 22722598 10.4161/auto.20339 PMC3429549

